# Transdermal Drug Delivery Systems and Their Use in Obesity Treatment

**DOI:** 10.3390/ijms222312754

**Published:** 2021-11-25

**Authors:** Zhiguo Li, Xuexun Fang, Dahai Yu

**Affiliations:** Key Laboratory for Molecular Enzymology and Engineering of Ministry of Education, School of Life Sciences, Jilin University, Changchun 130012, China; zgli20@mails.jlu.edu.cn (Z.L.); fangxx@jlu.edu.cn (X.F.)

**Keywords:** drug delivery, transdermal drug delivery, obesity, treatment

## Abstract

Transdermal drug delivery (TDD) has recently emerged as an effective alternative to oral and injection administration because of its less invasiveness, low rejection rate, and excellent ease of administration. TDD has made an important contribution to medical practice such as diabetes, hemorrhoids, arthritis, migraine, and schizophrenia treatment, but has yet to fully achieve its potential in the treatment of obesity. Obesity has reached epidemic proportions globally and posed a significant threat to human health. Various approaches, including oral and injection administration have widely been used in clinical setting for obesity treatment. However, these traditional options remain ineffective and inconvenient, and carry risks of adverse effects. Therefore, alternative and advanced drug delivery strategies with higher efficacy and less toxicity such as TDD are urgently required for obesity treatment. This review summarizes current TDD technology, and the main anti-obesity drug delivery system. This review also provides insights into various anti-obesity drugs under study with a focus on the recent developments of TDD system for enhanced anti-obesity drug delivery. Although most of presented studies stay in animal stage, the application of TDD in anti-obesity drugs would have a significant impact on bringing safe and effective therapies to obese patients in the future.

## 1. Introduction

Oral administration or injection are the two most widely used systemic drug delivery methods [1,2]. Small molecular drugs are mostly delivered through oral administration [3], while macromolecular drugs such as proteins or peptides are mainly given by injection [4,5]. Although oral administration is convenient, the drug availability is low, and some macromolecular drugs cannot be delivered. Injection can deliver macromolecular drugs, but it is not well accepted by patients because of its inconvenience and pain [6]. According to the survey, about 10% of adults have needle phobia [7]. Attention should also be paid to the risk of infection caused by the repeated use of needles [8]. The common disadvantage of systemic administration is that the blood concentration will decrease soon after administration, and it needs frequent administration to play a therapeutic role [9]. A large dose could, however, lead to many side effects. The development of new drug delivery methods is one of the hotspots in the field of medicine [10].

Transdermal administration has the advantage of no pain, no blood, easy self-administration, low drug dosage, and high drug integrity, which greatly reduces the adverse reactions caused by oral or injection drugs [3,11,12]. Drugs administrated through transdermal delivery enter the circulation slowly, avoid the first-pass effect of the liver, improve the curative effect, and reduce the frequency of drug administration [1,13,14]. Thus, it can be used for long-term self-administration, and it is also suitable for patients with inconvenient traditional administration such as coma or vomiting [15]. Transdermal administration can deliver most peptides or protein drugs that cannot be taken orally [3,16]. The comparison of drug delivery through the skin with oral administration and injection is shown in Table 1. In order to give full play to the advantages of transdermal drug delivery system (TDDS), continuous research is needed, to explore a variety of strategies to promote skin permeability. TDDS are being developed and studied in the treatment of obesity, Parkinsonism, diabetes, smoking cessation, analgesia, Alzheimer’s disease, hypertension, arthritis, osteoporosis, migraine, schizophrenia, and so on [17,18,19,20,21,22,23,24]. It is an excellent treatment especially for chronic diseases such as obesity that require long-term family treatment. We believe that TDDS may also become the mainstream form of anti-obesity drug delivery in the future. This paper briefly but comprehensively introduces the TDDS based on different strategies to promote skin permeability and some of its clinical applications. The side effects of drugs are not only related to their own properties, but also closely related to the mode of administration [25]. We summarize the administration methods and side effects of existing anti-obesity drugs and introduce several new types of anti-obesity agents under development. A comprehensive overview of TDDS for anti-obesity agents is also provided.

## 2. Strategies for Promoting Skin Permeability

The skin has a powerful barrier function, allowing only a very small number of lipophilic molecules to pass through [26,27]. The skin is mainly divided into three layers, epidermis, dermis, and subcutaneous tissue. The epidermis is the outermost layer of the skin, including the stratum corneum (SC). SC has extremely high density and hydrophobicity, which is the main obstacle to skin penetration [25,28,29]. The drug needs to pass through the epidermis to the dermis before it can work, and studies have found that there are three possible ways to do this, transcellular pathway, intracellular pathway, and appendage pathway (such as hair follicles, sebaceous glands, and sweat glands) [30,31]. The focus of the study of transdermal drug delivery technology (TDDT) is to enhance the permeability of drugs in the skin. At present, there are many studies on TDDT, and they use different methods to enhance the permeability of the skin in order to transport drugs into the dermis. It is mainly divided into passive enhancement and active enhancement [25]. we summarize the main contents of these two methods in detail here. Table 2 lists several drugs delivered through different transdermal delivery techniques.

### 2.1. Passive Promotion

The most common passive methods to improve skin permeability include the use of penetration enhancers (PE) and the use of nanocarriers to carry drugs into the skin [15,65,66,67,68].

PE can increase the diffusion and solubility of molecules in the skin, thus allowing molecules to infiltrate into the skin [69]. The main mechanism may be that PE destroys the corneal cell capsule, interacts with intracellular keratin, changes the composition of the SC and the partition coefficient between lipid bilayers [70,71]. There are many kinds of accelerators, including water, alcohols, fatty acids, peptides, sulfoxides, surfactants, terpene derivatives, etc. [15,72]. Atenolol as a β-adrenergic receptor blocking agent has been used to treat hypertension. Additionally, investigation shows that oral administration of the drug has side effects such as diarrhea, nausea, and colitis [73,74]. Cheong-Weon Cho et al. used ethylene vinyl acetate (EVA) as a transdermal absorption enhancer to promote the skin penetration of atenolol to treat hypertension. The results showed that the penetration enhancer improved the penetration of atenolol in isolated rat skin and alleviated the side effects of oral administration [32]. PE used for clinical administration should be tested for toxicity and hypersensitivity, and should have the characteristics of high permeation efficiency, controllable administration time and dose, and non-rejection with drugs. PE often irritate the skin, which limits their medical application to a great extent [75]. The research of PE has been going on for decades, and there is a more comprehensive review [15,30,76].

Nanocarriers can also infiltrate embedded drugs into the skin. They have been proved to be effective in the treatment of infection, cancer, hair loss, wound healing, and so on [69]. There are many kinds of nanocarriers, and their characteristics are different. This review briefly introduces the type, composition, structure, action mechanism, and application of nano-carriers.

#### 2.1.1. Liposomes

Liposomes are the most widely studied vesicular systems, mainly composed of cholesterol and phospholipids [77]. Liposomes have one or more bilayer lipid membranes for drug loading, with an average diameter of 50–300 nm [78,79,80]. Some studies indicate that the interaction between phospholipids and lipids in SC, brings molecules into the skin [81]. Studies have also shown that liposomes can also enter the skin through sebaceous glands [82]. The common preparation methods are film hydration, reverse phase evaporation, solvent injection, and so on [83,84,85]. Liposomes are non-toxic and have good biodegradability and can encapsulate hydrophilic and lipophilic molecules at the same time. Liposomes have been widely used in the treatment of acne and other skin diseases [86]. However, the fluidity of liposomes is low and the degree of skin penetration is not deep, so more studies are needed to enhance the skin permeability of liposomes [87].

#### 2.1.2. Ethosomes

Ethosomes are another new type of lipid carrier, which show superior drug delivery ability. Compared with ordinary liposome components, ethosomes add a higher concentration of ethanol (20–50%) [88]. Ethanol can increase the fluidity of cell membrane, destroy SC, and promote the role of drug delivery system (DDS) [89]. Ethosomes can carry both hydrophilic and lipophilic drugs and can deliver under occlusive conditions [26].

#### 2.1.3. Transferosomes

Transfersome is a highly elastic and overdeformed vesicle consisting of phospho-lipid bilayers and edge activators (such as sodium cholate, sodium deoxycholate) [90]. The edge activator makes transfersome have great flexibility and deformability, which can be passed between cells [91]. Notably, occlusive conditions inhibit skin penetration of transfersomes [92]. Hydrophobic molecules damage the elasticity of transporters, so they are not suitable for the delivery of hydrophobic drugs [93].

#### 2.1.4. Niosomes

Niosome is another vesicle that has emerged in recent years, which is composed of nonionic surfactants (such as polyoxyethylene alkyl ethers) and cholesterol [94]. The combination of nonionic surfactants and cholesterol changes the structure of SC, making it looser and more permeable [95]. Compared with ordinary liposomes, they have better stability and lower production cost.

#### 2.1.5. Glycerosomes

Glycerosomes are based on the addition of glycerol to ordinary liposomes [43]. The effect of glycerol is similar to edge activator, which increases the elasticity and deformability of the system and facilitates the penetration of the system through the skin [96].

#### 2.1.6. Lipid Nanoparticles

Lipid nanoparticles can be divided into solid lipid nanoparticles (SLN) and nanostructured lipid carriers (NLC), and mainly wrap drug molecules in non-aqueous cores [46]. SLN consists of solid lipids (such as triglycerides, fatty acids, steroids with diameters ranging from 50 to 1000 nm), surfactants (stabilization systems), and water [97]. NLC added liquid lipids on the basis of SLN to avoid the recrystallization of solid lipids and thus further improve the stability of the system [98]. The TDD mechanism of lipid nanoparticles is still under study. The greatest advantage of lipid nanoparticles lies in the sustainability of drug release, as well as their good biodegradability, compatibility, low toxicity, and production cost [68].

#### 2.1.7. Polymeric Nanoparticles

Both synthetic polymers and natural polymers can be used to prepare polymeric nanoparticles. Polymeric nanoparticles can produce drug concentration gradients to promote drug penetration [99]. Additionally, it has been shown that polymeric nanoparticles accumulate in the hair follicles and enter the skin along the hair follicles [100]. Chitosan is the most widely used positively charged natural polymer, which has good water solubility and bioadhesive properties [101]. Chitosan nanoparticles can be prepared by ionic cross-linking, precipitation, polymerization, and other methods, and the particle size ranges from 20 to 800 nm [68]. The connection between the drug and cationic chitosan is generally through electrostatic interaction, and the release time of the drug in the skin can range from days to months [102]. Natural polymers sometimes lack consistency between batches, which makes the preparation of natural polymer nanoparticles non-repeatable [103]. Different from natural polymers, synthetic polymers have good purity and consistency between batches, and are often used to transport hydrophobic drugs, such as poly (lactic-co-glycolic acid) (PLGA) [103,104]. PLGA has good biocompatibility and can encapsulate hydrophobic or lipophilic drugs, so it has been widely studied in local administration [105]. PLGA nanoparticles can be prepared by solvent replacement, solvent diffusion, phase transformation and other methods, and the particle size ranges from 10 to 1000 nm [68]. The problem of transdermal delivery by synthetic polymer nanoparticles is that volatile organic solvents are often used in the preparation of nanoparticles, and its residue will destroy the biological activity of peptides and protein drugs [106]. The preparation process of polymer nanoparticles needs to be continuously optimized.

#### 2.1.8. Metallic Nanoparticles

Nanoparticles prepared from gold, silver, titanium, zinc, and metal oxides can also be used as TDDS. Drug molecules are usually wrapped in the core of these particles, and these small nanoparticles have the ability to passively penetrate SC [107]. Studies have shown that the permeability is inversely proportional to the particle size [108]. Metallic nanoparticles have been widely studied because of their good biocompatibility, stability, easy preparation, and controllable shape [109]. The preparation methods include chemical synthesis, physical synthesis, photochemical synthesis, biosynthesis, and so on [110]. Gold nanoparticles are widely used in TDDS because of their low cytotoxicity and controllable particle size [111]. Some other metal particles have also been observed for transdermal applications. Silver nanoparticles (1–100 nm) have been shown to be effective in antibacterial and anti-inflammatory applications [112]. 

#### 2.1.9. Other

There are other nanocarriers, such as dendrimers, micelles, nanoemulsions, and nanogels. Dendrimers are composed of poly (amidoamine) and other polymers, which can be used to promote the skin penetration of hydrophilic drugs [113]. Micelles are composed of amphiphilic polymers or surfactants aggregated in aqueous media, which can carry both lipophilic and hydrophilic molecules [114]. Nanoemulsions are composed of surfactants, water, and oil, which can deliver a wide range of drug molecules [115]. Nanogels are a nano-sized cross-linked polymer, which can also be used for transdermal delivery of drugs [116].

### 2.2. Active Promotion

Active enhancement technology has also been advanced and widely used in recent years. Using them to deliver drugs is easier and faster to penetrate through the skin. The application of active enhancement technology is expanding, including magnetophoresis, iontophoresis, electroporation, and ultrasound to minimize invasive destruction of SC.

#### 2.2.1. Magnetophoresis

Magnetophoresis uses magnetic fields to promote the entry of drugs into the skin. The main principles may be that the magnetic field generated by the driving force could enhance the distribution of molecules in the SC [54]. Magnetophoresis has been shown to deliver drugs such as benzoic acid, salbutamol sulfate, and terbutaline sulfate through the skin [117]. S. Narasimha Murthy et al. designed a reservoir type transdermal patch system with amagnetic backing, and applied it to pig skin in vitro [54]. The result showed that the magnetic field could effectively enhance the permeation of lidocaine hydrochloride through pig skin. The magnetic field intensity was proportional to the drug release. The permeation flux of lidocaine hydrochloride was 0.53 ± 0.09 (μg/cm^2^/h) at 30 mT and 1.61 ± 0.12 (μg/cm^2^/h) at 300 mT. Magnetic fields of different intensities (30–300 mT) were generated by changing the distance between the poles of the magnets or using magnets of different intensities. In rats, the skin bioavailability of the magnetic patch system was also significantly higher than that of the non-magnetic control patch. The disadvantage is that the system is not portable, and more cumbersome to use.

#### 2.2.2. Iontophoresis

Iontophoresis creates a driving force on charged molecules into the skin through electrostatic effects by disrupting the order of lipids in cells and increasing the fluidity of keratin structures [118]. The amount of drug delivered is proportional to the applied current, and the amount of drug delivery can be well controlled [25,119]. In general, it does not exceed 0.5 mA/cm2 [120]. Iontophoresis has been used in the delivery of drugs such as fentanyl, lidocaine, steroids [55,56,57]. The efficiency of iontophoresis depends on the physical and chemical properties of the molecule such as molecular weight, charge, polarity, valence state, structure, degree of dissociation, etc. Studies have shown that the degree of dissociation is the key factor in the efficiency of iontophoresis [119]. The current used for iontophoresis includes direct current (DC), alternating current (AC), and pulse [56]. DC iontophoresis is the most widely studied TDDT. Its disadvantage is that after application the skin will produce discomfort, and even burns [121]. AC iontophoresis and pulsed iontophoresis produce poor driving force effects, but can avoid skin discomfort to some extent [122]. Previous study used the DC iontophoresis technique (0.3 mA/cm^2^, 8 h) to deliver the functional protein ribonuclease noninvasively into isolated pig skin [118]. The results showed that molecular cumulative permeability is 224.37 ± 72.34 µg/cm^2^ and the function of ribonuclease is realized. There are still many problems in the wide application of iontophoresis technology in clinic, and further research needs to be conducted in the areas such as the exploration of miniaturization of equipment, the improve of efficiency of iontophoresis in delivering uncharged molecules, the revelation of the relationship between iontophoresis efficiency and physicochemical properties of drugs, and the clarification of specific mechanism of iontophoresis to improve transdermal efficiency.

#### 2.2.3. Electroporation

Electroporation is a method to improve the permeability of cell membrane instantly by high intensity electric field [123]. The increase of permeability may be due to the temporary water holes on the cell membrane caused by pulsed electric field [124]. Electroporation generally has two pulse types, exponential decay pulse and square wave pulse. The applied voltage ranges from 50 to 1500 V, the duration is 10 μs–10 ms, and the pulse interval can range from seconds to minutes [117,123]. The electrical parameters can be continuously adjusted and optimized according to the clinical situation. In general, the penetration efficiency is proportional to the pulse amplitude and duration [125]. Electroporation has been proved to be effective in the delivery of proteins and nucleotides and has been used to deliver drugs such as fentanyl, insulin [126,127]. Electroporation has great potential in the treatment of cancer, infectious diseases, and vaccination. Although its specific mechanism is not clear, the application of electroporation TDDT will be more and more frequent in the future. It is worth noting that electroporation may stimulate nerves to cause pain or muscle contraction and poor repeatability of drug delivery doses, which are the primary solutions for large-scale clinical applications [123,128,129].

#### 2.2.4. Ultrasound

Drugs can be introduced into the skin by ultrasound, to achieve systemic or local administration. The main mechanism of ultrasonic penetration of SC is that the cavitation effect produces voids in the skin, resulting in an increase in tissue permeability [117]. At present, the application and research of ultrasound include low frequency ultrasound (LFS) introduction (20–100 kHz) and high frequency ultrasound (HFS) introduction (0.7–16 MHz). It is found that the cavitation effect is inversely proportional to the ultrasonic frequency, so LFS can promote the penetration of drug molecules more effectively [130]. LFS can deliver a variety of molecules, including hydrophobicity, hydrophilicity, low molecular weight, and high molecular weight drugs. Drugs such as lidocaine, dexamethasone, and ibuprofen have been reported to be delivered by ultrasound [59,60,61]. The enhancement of skin penetration efficiency and the portability of ultrasound equipment are of great significance for the wider clinical application of ultrasound.

#### 2.2.5. MNS

**Figure 1:**
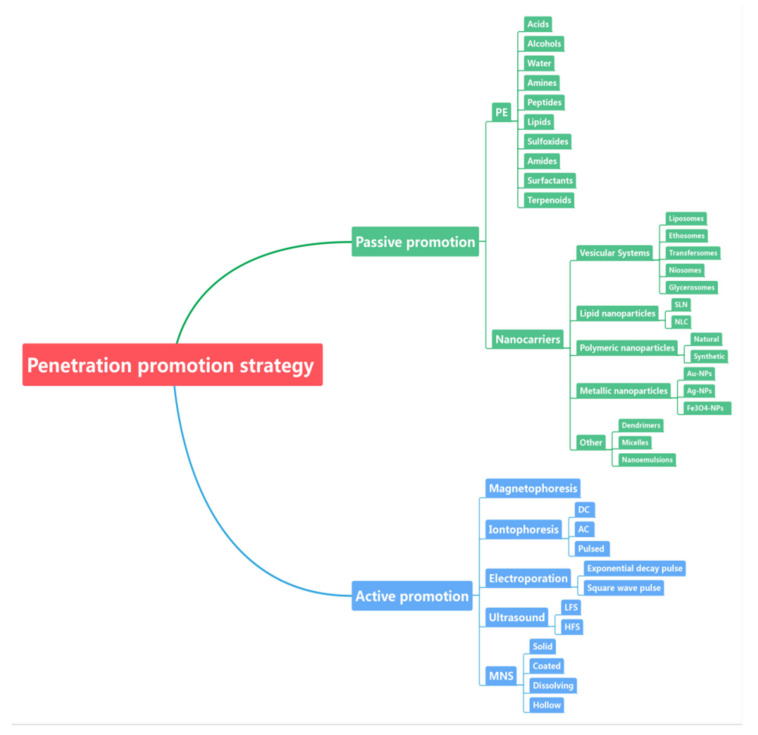
The type of penetration promotion strategy.

Microneedles (MNS) with minimally invasive destruction of SC have been the most widely used TDDT. MNS use micron needles, mostly 150–1500 microns long and 50–250 microns wide, with a tip thickness of 1–25 microns, to penetrate only the upper layer of the skin, with no direct contact with nerve fibers and blood vessels [131]. This painless drug delivery technique can also reduce the risk of infection after use [12,132,133,134]. MNS are often designed as microneedle arrays (MNA), which combine the advantages of transdermal patches and subcutaneous injection. There are many types of MNS, such as solid, coated, dissolving, and hollow MNS. Solid MNS first destroys the SC, then a drug patch is applied to the treatment site; the coated MNS coated the drug on the MNS and inserted it into the skin; the dissolving MNS are a soluble MNS containing drugs; the hollow MNS contain drugs that can be injected into the skin [132]. Various types of materials, such as polymers, metals, or silicon can be used to make MNS. Common materials are hyaluronic acid (HA), polyglycolic acid (PGA), PLGA, and carboxymethyl cellulose (CMC) [117]. Hye-Rin Jeong et al. prepared a dissolving MNS containing cyclosporine A (600 μm in length and 250 μm in width) [135]. The system (containing 50% cyclosporine A) was put into pigskin for 60 min. The results showed that 65% of MNS length was dissolved and about 34 μg cyclosporine A were injected into the skin. Clinical studies have shown that MNS can deliver many drugs, including fentanyl, insulin, lidocaine, and vaccines [62,136,137,138]. At present, MNS technology is the most potential and widely studied technology to actively enhance skin permeability. The advantages of microneedle technique include fast onset, strong patient compliance, obvious effect, and good repeatability (the dosage can be controlled) [14]. On the other hand, some shortcomings of microneedle technique are also worthy of attention, such as skin irritation, infection, limited drug loading, and so on. The development direction of this technology is to produce MNS with sufficient mechanical strength at minimum cost, increase drug loading on MNS, and expand the transport path to facilitate the passage of macromolecules through the skin. The type of penetration promotion strategy is summarized in Figure 1.

## 3. The Main Delivery Methods of Anti-Obesity Drugs and Development of New Anti-Obesity Agents

### 3.1. The Main Delivery Methods of Anti-Obesity Drugs

Obesity directly or indirectly causes hundreds of diseases, including central nervous system diseases such as depression and Alzheimer’s disease [139,140,141]; respiratory diseases including chronic bronchitis and emphysema [142,143]; cardiovascular diseases including atherosclerosis and hypertension [144]; digestive diseases including fatty liver and ulcerative colitis [145,146]; metabolic diseases including diabetes and gout [147,148]; bone and joint diseases including osteoporosis and arthritis [149]. Obesity may also lead to kidney disease, male sexual dysfunction, irregular menstruation, and even infertility in women [150,151]. In addition, obesity is also a well-known cause of many cancers, including liver cancer, colorectal cancer, stomach cancer, breast cancer, endometrial cancer, and so on [152,153,154]. Obesity patients have increased incidence of inflammation, low immunity, and accelerated aging [155]. It is necessary for obese patients with body mass index (BMI) > 27 kg/m^2^ and other complications to be treated with medication. 

Oral or injection is still the main delivery mode of anti-obesity drugs. There are five main types of anti-obesity drugs approved by the US Food and Drug Administration (FDA): Orlistat, Phentermine/Topiramate ER, Naltrexone SR/Bupropion SR, Lorcaserin and Liraglutide 3.0 mg. The effectiveness of these drugs is only 3–7% by the present delivery mode. Liraglutide 3.0 mg is administered by injection, and all other drugs are administered orally at present [156,157]. 

The main mode of action of Phentermine/Topiramate ER, Naltrexone SR/Bupropion SR, Lorcaserin and Liraglutide 3.0 mg is to control appetite and enhance satiety to reduce calorie intake. Additionally, the exact mechanism is still being studied [158]. These drugs have more or less some adverse reactions, including elevated blood pressure, arrhythmia, dizziness, insomnia, nausea, taste failure, constipation, and so on. Thus, the use of these drugs is often prohibited for patients with cardiovascular diseases, people taking other drugs, and women during pregnancy [159]. Orlistat is a lipase inhibitor that blocks the body’s absorption of fat, reduces calorie intake, and controls weight [156]. Orlistat is the only anti-obesity drug approved by China’s State Food and Drug Administration. It is the best-selling over the counter (OTC) anti-obesity drug in the world and can be taken by teenagers [160]. Although the drug is very popular in weight loss, it also has some side effects, such as diarrhea, flatulence. Metformin has also been shown to have a weight-loss effect. Metformin is the first oral drug of choice for obese patients with type 2 diabetes and known side effects include stomachache and constipation [161,162,163].

Obviously, there are many disadvantages for these drugs to enter the human body through the traditional oral or injection way, such as low efficacy, some side effects, large dose, poor patient compliance, inconvenient use, and so on. Patients need to understand the side effects of these drugs before deciding whether or not to use them. Some oral anti-obesity drugs that were once approved by FAD are now banned. In early 2020, FDA issued a warning that weight loss drugs Belviq and BelviqXR (lorcaserin) may increase the incidence of cancer in patients, but it is unclear whether the drug will be banned [164]. Table 3 lists the known principles, common delivery methods, and some side effects of some anti-obesity drugs.

Systemic drug delivery is considered to be one of the main causes of various side effects, which may be caused by large doses and limit drug availability to a great extent [25]. Therefore, in order to reduce drug dosage, reduce drug side effects, avoid needle fear of patients, avoid infection and continuous weight loss, constantly developing new anti-obesity drugs, drug delivery methods also need to be further studied.

### 3.2. Development of New Anti-Obesity Drugs

Obesity is actually the result of an imbalance between energy intake and consumption. Too much energy intake that cannot be consumed in time will be stored by adipocytes [165]. In addition to reducing appetite, increasing satiety, and inhibiting the absorption of nutrients, the anti-obesity drugs currently being studied focus on promoting fat decomposition or adipocyte heat production [166,167]. Adipose tissue (AT) is mainly distributed in the subcutaneous and visceral organs. Subcutaneous AT stores more than 80% of human body fat, and reducing the amount of local subcutaneous AT is very helpful for the treatment of obese patients [168]. Adipocyte tissue can be divided into brown adipocyte tissue (BAT) and white adipocyte tissue (WAT) [169]. The main function of WAT is to store energy, and WAT, which is one of the causes of many complications of obesity, is usually too high in obese patients [170]. Unlike WAT, BAT contains metabolically active brown fat cells that generate heat and increase the body’s energy consumption. As a thermogenic protein, uncoupling protein 1 (UCP1) is the main protein mediating this process. Taking WAT as an anti-obesity target transforming it into brown-like adipocytes (browning) and increasing human energy consumption is considered to be a very promising way to achieve anti-obesity [171,172,173]. It has been proved that several substances can promote browning to increase thermogenesis, such as β3-adrenoceptor agonist, thyroid hormone T3, rosiglitazone (ROSI), bile acid, fucoxanthin, curcumin, and so on [165,174,175,176]. Gelatin, gold nanoparticles, and caffeine can promote fat decomposition [177,178,179]. It was found that glucagon-like peptide 1 (GLP1) analogue and resveratrol could not only activate brown adipocytes to enhance thermogenesis, but also promote browning [180,181,182]. Table 4 lists some of the new anti-obesity agents being studied, as well as their possible mechanisms of action and their delivery methods.

## 4. TDDS for Anti-Obesity Agents

People generally have a low level of awareness of obesity, and they think that there is no need for drug treatment or understand the side effects of drugs, resulting in resistance to long-term medication. Therefore, the development of external anti-obesity drugs with low side effects may have more extensive research and application prospects. TDDS has the advantages of low dose, high bioavailability, low side effects, and easy application, so it is very suitable for delivering anti-obesity drugs. The high targeting of TDDS also seems to show great potential for the reduction of local subcutaneous AT [197]. In order to improve the role of agents in anti-obesity and avoid the adverse effects caused by defects in the drug delivery system, some scholars began to study the transdermal delivery of anti-obesity agents.

### 4.1. Transdermal Delivery of Common Browning Agents

**Figure 2:**
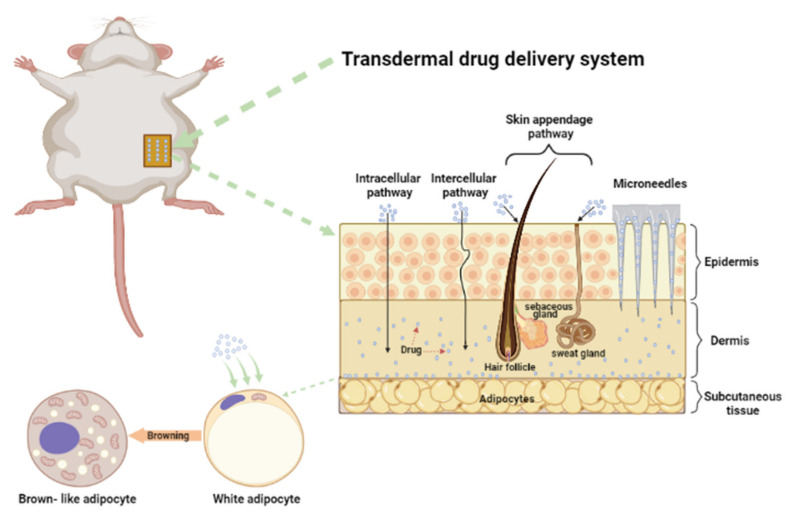
TDDS for managing obesity.

TDDS for managing obesity is illustrated in Figure 2. CL316243, a β3-adrenoceptor agonist, has been shown to promote the browning of adipocytes in obese mice [183]. FDA has approved Mirabegron, a β3-adrenoceptor agonist for the treatment of overactive bladder. However, systemic use of the drug can lead to an increase in heart rate and blood pressure [198]. Thyroid hormone T3 can promote fat browning and increase thermogenesis. Human and animal experiments have shown that T3 or T4 can lead to weight loss [199,200]. The reason why thyroid hormone has not been approved as an anti-obesity drug is that long-term systemic use of T3 can lead to hyperthyroidism and cardiovascular disease [201]. Peroxisome proliferator-activated receptor γ (PPARγ) is not only the main gene regulating fatty acid storage and glucose metabolism, but also one of the main transcriptional regulators of BAT production [202]. ROSI is a kind of PPARγ activator, which can enhance the sensitivity of skeletal muscle, liver, and AT to insulin, and it has been used to treat diabetics [187]. Recent studies have shown that it also has the effect of browning [175]. However, taking ROSI also has a potential risk of cardiovascular disease [203].

AungThan et al. made a fast-dissolving MNS based on HA. The drug can enter the skin in two minutes. They designed an animal experiment that quickly delivered β3-adrenoceptor agonist (CL316243) and thyroid hormone (T3) to subcutaneous WAT [186]. They placed the MNS patch on the groin of diet induced obese (DIO) mice. The results after 5 days of treatment showed that the CL316243 released by MNS could effectively promote the browning of WAT cells and inhibit weight gain. Transdermal administration of T3 also inhibited weight gain without systemic hyperthyroidism. Compared with the intraperitoneal injection group, the dose of transdermal administration of the two drugs was lower (only when the injection dose was increased by 5 times, the effect of fat reduction was similar to that of MNS injection), the specificity was high, and almost no side effects were observed. No obvious skin damage was observed after administration, which proved that the MNS patch was safe to use in mice. The food intake of mice in the treatment group was basically the same as that in the control group. In the treatment group, weight of the epididymis WAT (epiWAT) and the total weight of inguinal WAT (igWAT) on the patch side were significantly decreased, and the total weight of igWAT on the non-patches side was also decreased to some extent. This result shows that the percutaneous release of browning agent can not only target the treatment of local obesity, but also reach other AT through skin circulation.

Obesity requires long-term and frequent administration of anti-obesity drugs. Thus, the development of sustainable anti-obesity drugs is very promising. Yang, H et al. further developed a slowly dissolving PLGA-MNS-patch. PLGA is also a microneedle polymer with excellent biocompatibility and biodegradability [204]. The fluorescent Cy5 molecules were evenly distributed in the MNS patch, and the sustained release effect of the drug delivery was observed. The results showed that the fluorescence signal was retained at the insertion site for 5 days, indicating that PLGA-MNS has a very good sustained release effect. MNS sustained-release patches with CL316243 were used to treat DIO mice. Compared with the non-drug patches, the five-day treatment inhibited about 15% of the weight gain of the mice. However, intraperitoneal injection of the same dose of CL316243 could not significantly inhibit the weight gain of mice. It was also observed that the body temperature and the expression of UCP1 in adipose tissue increased in the MNS treatment group, which confirmed that transdermal delivery of CL316243 promoted the browning process. The food intake of mice in each group also had no significant difference, and the use of MNS would not cause obvious skin abnormality. Finally, the detection of serum biochemical indexes in mice showed that some indexes of metabolic syndrome such as total cholesterol, free fatty acids, and insulin decreased after MNS treatment.

Yuqi Zhang et al. designed a NPs-based HA-MNS patch that can transport NPs loaded with ROSI or CL 316243 to the groin of mice [188]. They first studied it in thin mice. The mice were divided into three groups and were given three kinds of MNS patch: HA-MNS patch, HA-MNS patch with Rosi NPs, and HA-MNS patch with CL316243 NPs. The interval period for the use of the patch was 3 days. After 6 days of treatment, the inguinal AT of mice was removed. The results showed that the number of beige adipocytes in ROSI- NPs-MNS group and CL 316243-NPs-MNS group increased, UCP1 gene upregulated, while IL-6 gene decreased. Together, they proved that the browning agent delivered by MNS patch effectively promoted the browning process of WAT. Meanwhile, it was observed that there was no significant change in food intake and oxygen consumption in the two treatment groups. After that, they further studied the therapeutic effect of MNS browning agent patches on obese DIO mice. In the treatment group, ROSI- NPs-MNS patches or CL316243-NPs-MNS patches were placed in one groin area of DIO mice, and HA-MNS patches were placed on the other side. For the control group, HA-MNS patches were placed on both sides. The results showed that the treatment group inhibited about 15% weight gain and reached 30% reduction in epiWAT. HE staining showed obvious browning only in the adipocytes near the drug-containing patch, which proved that the browning agent delivered by HA-MNS patches promoted the local browning of WAT.

Yixuan Xie et al. designed a biodegradable MNS patch based on PLGA and polylactic acid (PLA) [205]. The degradation products of PLGA are harmless lactic acid and glycolic acid. The hardness of MNS can be increased by adding PLA [206]. Then, they used DIO mice as experimental subjects to study the effect of MNS patches on delivering browning agent CL316243. The experimental treatment groups included CL316243 MNS group (0.1 mg/kg/day) and injection group (1 mg/kg/day). After 15 days of administration, there was no significant change in the food intake of mice, but the body weight of mice in the two treatment groups became lighter. It was found that the total weight of BAT organs increased by about 36% and that of igWAT decreased by about 42%. The expression of UCP1 in AT of mice was detected by Western blotting, and the expression of UCP1 in the treatment group was also significantly increased. It is worth noting that the dose of MNS in the treatment group was only 1/10 of that of injection, but the therapeutic effect was as significant as that of injection.

Young Hyeon An et al. made a disposable portable iontophoresis system using reverse electrodialysis (RED) technology [207]. The system cooperates with polypyrrole-polyvinyl (PYP) alcohol-based conductive hydrogel for drug delivery. PYP hydrogel promotes electron transport and accelerates the mobility of electrically movable drug nanocarriers (DNS) embedded in PYP hydrogel. Ionizable DNS can improve the drug delivery efficiency of iontophoresis. They further studied the therapeutic effect of the system transdermal delivery of ROSI on diet-induced type 2 diabetes and obese mice. ROSI was encapsulated in electrically removable DNS, and the system was applied to the right inguinal region of obese mice by cathodic iontophoresis. After 4 weeks of treatment, the level of blood glucose decreased significantly and the body weight decreased by about 12%. After dissection, it was found that the size of AT was significantly reduced in the treatment group. Histological analysis showed that obvious browning was observed at the site of administration. In the end, they also conducted a skin damage test and found that the system did not irritate the skin and did not cause inflammation in the skin tissue.

### 4.2. Transdermal Delivery of Curcumin

Nanofibers increase the diffusion rate and uniform distribution of drugs on the surface of the skin [208]. Ariamoghaddam AR et al. designed a TDDS of polyvinyl alcohol-gelatin nanofibers with a diameter of 200–250 nm, through which curcumin was delivered to rats to treat obesity [190]. Curcumin is a compound extracted from traditional edible fungi, which is considered to have the effects of anti-inflammation, weight loss, anti-cancer, and so on [209]. Some studies have shown that the anti-obesity effect of curcumin might be caused by browning [189]. The release rate of curcumin reached more than 50% after 20 h. Another reason for the rapid diffusion may be the existence of gelatin, which increases the solubility of curcumin and obtains higher surface utilization. It is estimated by whole-body magnetic resonance imaging that the total number of AT in rats is reduced by 4% and 7% when administered in this way.

### 4.3. Transdermal Delivery of Gold Nanoparticles

Gold nanoparticles (AuNPs) have been shown to be used as anti-obesity drugs, which can absorb visible light and near infrared (NIR) light. The light can be converted efficiently by surface plasmon resonance (SPR) [178]. Jung Ho Lee et al. developed hyaluronate-hollow gold nanosphere-adipocyte targeting peptide (HA-HAuNS-ATP) conjugates, which can be used for photothermal decomposition of lipids [196]. HA can enhance the stability and biocompatibility of HAuNs. HAuNs have better photothermal properties. Adipocyte targeting peptide (ATP) with specific sequences can improve the targeting of the system to AT. First of all, cytotoxicity experiments show that the system will not cause serious damage to cells. The results of PA imaging confirmed that HA-Hauns-ATP conjugates had an effective transdermal penetration effect on the abdominal skin of mice. Then they used conjugates to treat obese mice fed with high fat diet (HFD) and observed the photothermal decomposition of fat. After DIO mice were anesthetized, HAuNS, HA-AuNS conjugates and HA-HAuNS-ATP conjugates were applied to the abdominal skin of mice. They wiped off the remaining drugs in 1 h, irradiated NIR light on the drug site for 10 min, and finally analyzed the photoacoustic imaging of PA imaging. The results showed that under NIR irradiation, HA-HAuNS-ATP conjugates could better decompose the AT of obese mice, reducing fat by about 20%, and there was no skin damage after treatment.

### 4.4. Transdermal Delivery of Gelatin

Some natural polymers (such as gelatin, chitosan) are widely used as drug carriers, but there are reports of increased levels of glycerol released by adipocytes treated with natural polymers, which means lipolysis occurs [210]. Oral natural polymers have a great disadvantage in reducing subcutaneous fat because of their low administration efficiency. Sung-Min An et al. reported a method of using microneedles to assist transdermal delivery of gelatin [177]. They carried out animal experiments with dissolving microneedle (DMN) patches made from gelatin. Gelatin microneedle (GMN) patches were applied to the back skin of obese rats induced by HFD for 2 days. At the end of the experiment, the fat under the MNS was taken for analysis. The results showed that the area of subcutaneous fat in the treatment group was significantly reduced compared with the obese rats after using GMN patches. 

### 4.5. Transdermal Delivery of Caffeine

Caffeine is a natural ingredient in tea and coffee, which has been proved to have anti-obesity activity. The availability of oral caffeine is low, and the blood concentration decreases quickly after administration. Manita Dangol et al. reported a new type of caffeine percutaneous DMN patch and observed its anti-obesity effect on DIO mice [197]. In biofilms, caffeine is difficult to transmit through the skin because the multiform transition from anhydrous to aqueous leads to crystal growth. Polymers such as HA can maintain the anhydrous form of caffeine and inhibit crystal growth, so they designed a DMN based on HA to deliver caffeine. DIO mice in the treatment group were smeared with DMN on the back three times a week for 6 weeks. After administration, it was concluded that there was no significant change in the food intake of obese mice in each group, and the body weight of mice in the treatment group decreased by about 13%. The levels of serum triglyceride (TG) and total cholesterol (TCHO) and other biochemical indexes of obesity in DIO mice also decreased significantly, which confirmed that the system has a good effect on anti-obesity in DIO mice.

## 5. Conclusion and Prospect

TDDS increases the targeting and availability of drugs and shows many unique advantages in the delivery of new anti-obesity drugs, especially in promoting local fat decomposition or transformation. In addition to the toxicity of anti-obesity drugs, the oral and injection mode of drug delivery could cause serious side effects. The anti-obesity drug through TDDS has the advantages of low dose, less side effects, and easy to use, which can bypass the shortcomings of systemic administration. This paper introduces the research progress of several new anti-obesity drugs delivered through TDDS. Animal experiments have proved that these TDDS promote anti-obesity effects, and have obvious advantages compared with systemic drug delivery. As obesity requires long-term family treatment, some TDDS with continuous treatment are being studied. The effect of TDDS on the skin also needs further attention. Some penetration promoting factors would cause adverse reactions to the skin, so the skin permeability and biocompatibility need to be considered in the construction of the TDDS to release or eliminate skin discomfort. Allergenicity also needs comprehensive investigation in further research. The dose of new anti-obesity drugs delivered by TDDS can be adjusted by the strength of applied current, the size of magnetic field, the shape or number of microneedle patches, which expands the scope of application and conditions of TDDS in obesity treatment. MNS technology is developing rapidly, especially the new biodegradable microneedles (BMN). For chronic diseases like obesity, BMN is the most promising TDDS due to its convenience, safety, low dose, and low cost.

At present, drugs through transdermal administration to treat human obesity have not been reported. In general, TDDS for the treatment of human obesity still needs further study to investigate the effectiveness of drugs on the human body, long-term use safety, efficacy sustainability, ease of use and appropriate dosage, and so on. In the meantime, drug delivery carriers also need to be evaluated on their safety, cost, and industrial production. The research on these aspects is the focus of the application of TDDS in the treatment of human obesity in the future. However, there is an urgent need to fully understand the advantages and disadvantages of these formulations and to overcome challenges for clinical translation.

## Figures and Tables

**Table 1:** The comparison of drug delivery through the skin with oral administration and injection.

Drug Administration Route	Range	Advantage	Deficiency	Types
Injection	Whole body	Can deliver macromolecular drugs such as proteins or peptides Quick curative effect	Needle phobia Infected Family therapy is almost impossible Serious side effects Low targeting	Intravenous injection Hypodermic injectionIntramuscular injection Intraperitoneal injection
Oral		Easy to use and can be treated at home for a long time High compliance of patients	Low bioavailability of drugs Serious side effects Unable to delivermacromolecular drugs Low targetingHigh dose	Capsule Pills Granule Oral liquid
Transdermal	Whole body, Local	Easy to use and can be treated at home for a long time High compliance of patients Sustainable drug delivery High targeting High bioavailability of drugs Little side effect Low dose	Some systems have low transdermal efficiency May irritate the skin Need to continue to study the security of long-term use	Patch Ointment Coating agent

**Table 2:** Examples of drugs delivered by different transdermal delivery techniques.

The Form of Promoting Infiltration	TDDT	Drugs	References
Passive	PE	Tetracaine, Atenolol, Diltiazem hydrochloride	[32,33,34]
	Ethosomes	ketoprofen, Vancomycin Hydrochloride	[35,36]
	Liposomes	Indinavir, Propranolol	[37,38]
	Transfersomes	Raloxifene Hydrochloride, Itraconazole,	[39,40]
	Niosomes	Simvastatin, Buflomedil Hydrochloride	[41,42]
	Glycerosomes	Diclofenac Sodium, Celecoxib	[43,44]
	Lipid NPs	Ivermectin, Olanzapine	[45,46]
	Polymeric NPs	Repaglinide, Agomelatine	[47,48]
	Au-NPs	Methotrexate	[49]
	Ag-NPs	Donepezil	[50]
	Dendrimers	Endoxifen	[51]
	Micelles	Insulin	[52]
	Nanoemulsions	Lidocaine	[53]
Active	Magnetophoresis	Lidocaine	[54]
	Iontophoresis	Fentanyl, Lidocaine, Steroids, Sumatriptan	[55,56,57]
	Electroporation	Insulin, DNA vaccine	[16,58]
	Ultrasound	Lidocaine, Dexamethasone, Ibuprofen	[59,60,61]
	MNS	Insulin, Naltrexone, Propranolol	[62,63,64]

**Table 3:** Known principles, common delivery methods, and side effects of common anti-obesity drugs.

Drug Name	Known Principle of Action	Delivery Mode	Side Effect
Phentermine /Topiramate ER	Reduce appetite	Oral administration	Insomnia, constipation, dizziness, taste disorders
Naltrexone SR/Bupropion SR			Diarrhea, constipation, headache
Lorcaserin			Dizziness, constipation, nausea
Rimonabant (delisted)			Nausea, gastrointestinal discomfort
Fenfluramine (delisted)			Heart valve damage, hypertension
Liraglutide 3.0 mg		Hypodermic injection	Depression, dizziness, neuropsychiatric diseases
Orlistat	Block the absorption of fat	Oral administration	Diarrhea, flatulence
Sibutramine (delisted)	Reduce appetite, increase energy consumption		Cardiovascular and cerebrovascular diseases

**Table 4:** Possible action principle and delivery methods of new anti-obesity agents.

Agent Name	Known Principle of Action	Delivery Mode	References
β3-adrenoceptor agonist (CL316243)	Browning and A=activate brown adipocytes	Hypodermic injection, transdermal	[183,184]
Thyroid hormone (T3)			[185,186]
ROSI		Oral administration, transdermal	[187,188]
Curcumin			[189,190]
Fucoxanthin		Oral administration	[191]
Bile acid			[192]
Capsaicin			[193]
Olive oil			[194]
GLP1 analogue		Hypodermic injection	[180]
Resveratrol		Oral administration	[182]
Gelatin	Fat decomposition	Oral administration, transdermal	[177]
Caffeine			[179,195]
Gold nanoparticles		Transdermal	[178,196]

## Abbreviations

AT	Adipose tissue
AC	Alternating current
ATP	Adipocyte targeting peptide
AuNPs	Gold nanoparticles
BAT	Brown adipocyte tissue
BMI	Body mass index
BMN	Biodegradable MN
CMC	Carboxymethyl cellulose
CPE	Chemical penetration enhancer
DC	Direct current
DIO	Diet-induced obese
DMN	Dissolved MN
DMSO	Dimethyl sulfoxide
DNS	Drug nanocarriers
EpiWAT	Epididymis WAT
EVA	Ethylene vinyl acetate
FDA	Food and drug administration
GLP1	Glucagon-like peptide 1
GMN	Gelatin microneedle
HFD	High fat diet
HFS	High frequency ultrasound
ICU	Intensive care unit
IgWAT	Inguinal WAT
IL-6	Interleukin- 6
LFS	Low-frequency ultrasound
MNA	Microneedle arrays
MNS	Microneedles
NLC	Nanostructured lipid carriers
NIR	Near infrared
NPs	Nanoparticles
OTC	Over the counter
PA	Photoacoustic
PE	Penetration enhancers
PGA	Polyglycolic acid
PLA	Polylactic acid
PLGA	Poly (lactic-co-glycolic acid)
PPARγ	Peroxisome proliferator-activated receptorγ
PTH	Parathyroid hormone
PYP	Polypyrrole-polyvinyl
RED	Reverse electrodialysis
ROSI	Rosiglitazone
SC	Stratum corneum
SLN	Solid lipid nanoparticles
SPR	Surface plasmon resonance
TCHO	Total cholesterol
TDD	Transdermal drug delivery
TDDS	Transdermal drug delivery system
TDDT	Transdermal drug delivery technology
TG	Triglyceride
UCP1	Uncoupling protein 1
WAT	White adipocyte tissue
